# A novel hybrid methodology to secure GOOSE messages against cyberattacks in smart grids

**DOI:** 10.1038/s41598-022-27157-z

**Published:** 2023-02-01

**Authors:** Shahbaz Hussain, Atif Iqbal, S. M. Suhail Hussain, Stefano Zanero, Abdullatif Shikfa, Enrico Ragaini, Irfan Khan, Rashid Alammari

**Affiliations:** 1 Department of Electrical Engineering, Qatar University, Doha, Qatar; 2 Dipartimento di Elettronica, Informazione e Bioingegneria, Politecnico di Milano, Milan, Italy; 3 Department of Electrical Engineering, King Fahd University of Petroleum and Minerals (KFUPM), Dhahran, Saudi Arabia; 4 Interdisciplinary Research Center for Renewable Energy and Power Systems (IRC-REPS), King Fahd University of Petroleum and Minerals (KFUPM), Dhahran, Saudi Arabia; 5 College of Computing and Information Technology, University of Doha for Science and Technology, Doha, Qatar; 6 Department of Electrical and Computer Engineering, Texas A&M University, Texas, USA

**Keywords:** Engineering, Electrical and electronic engineering

## Abstract

IEC 61850 is emerging as a popular communication standard for smart grids. Standardized communication in smart grids has an unwanted consequence of higher vulnerability to cyber-attacks. Attackers exploit the standardized semantics of the communication protocols to launch different types of attacks such as false data injection (FDI) attacks. Hence, there is a need to develop a cybersecurity testbed and novel mitigation strategies to study the impact of attacks and mitigate them. This paper presents a testbed and methodology to simulate FDI attacks on IEC 61850 standard compliant Generic Object-Oriented Substation Events (GOOSE) protocol using real time digital simulator (RTDS) together with open-source tools such as Snort and Wireshark. Furthermore, a novel hybrid cybersecurity solution by the name of sequence content resolver is proposed to counter such attacks on the GOOSE protocol in smart grids. Utilizing the developed testbed FDI attacks in the form of replay and masquerade attacks on are launched and the impact of attacks on electrical side is studied. Finally, the proposed hybrid cybersecurity solution is implemented with the developed testbed and its effectiveness is demonstrated.

## Introduction

With the amalgamation of information and communication technologies (ICT) in power grids, the traditional power systems are rapidly evolving as smart grids. ICT enables remote monitoring, control, and automation of power systems^1^. For interoperable operation of smart grids many communication protocols and standards are proposed. Among them IEC 61850 has emerged as one the most popular and widely accepted standard for power utility systems^2^.

Standardized communication and protocols in smart grids present an increased vulnerability to cyber-attacks. The attackers may exploit the standardized semantics to launch different types of attacks on standardized communication. IEC 61850 communication protocols are vulnerable to cyber-attacks. In literature, many attacks on generic object-oriented substation event (GOOSE) and sampled value (SV) messages are widely reported^3,4^. Previous studies in literature showed that GOOSE messages are most vulnerable, a single contaminated GOOSE message can result in successful maloperation of circuit breakers and result in severe consequences^5,6^. IEC 61850 standard does not present any considerations or strategies for GOOSE messages against cyberattacks. IEC 62351 standard series compliments IEC 61850 standard by providing cybersecurity considerations for different IEC 61850 messages^7^.

In literature, researchers focused on developing information technology (IT) or operational technology (OT) based solutions for securing GOOSE messages against different attacks. For instance, in^8,9^ authors proposed use of Rivest–Shamir–Adleman (RSA), elliptic curve digital signature algorithm (ECDSA) and rainbow signature scheme (RSS) based digital signatures for securing GOOSE messages. However, in^8,10^ it was proved that the digital signatures result in high computational delays and hence not suitable for time critical GOOSE messages with stringent 3 ms timing requirements. Recently published IEC 62351-6 standard proposed light weight message authentication code (MAC) algorithms to secure the GOOSE messages^11–13^. Authors in^14^ introduced caching-based MAC and less-online/more-offline MAC signatures which further reduces computational delays. Although the MAC algorithms have very less computational delays, they are symmetric algorithms which require a pre-shared key. Safe distribution and update of pre-shared keys is a quite challenging and in turn requires robust key distribution mechanism.

On the other hand, OT based solutions (generally outside the IT domain) for securing GOOSE message against cyber-attacks were developed. In such solutions, the contents of the communication messages are verified before they are processed further. This verification can be carried out by various methods, such as confirming the message contents received by the neighboring IEDs^15^, or using machine learning tools to detect abnormal GOOSE messages^16^. In^17^, authors presented a sliding window-based sequential classification mechanism to detect abnormalities. Similarly, in^18^, discrete wavelet transform (DWT) and Long Short-Term Memory (LSTM)-based autoencoder network is proposed to detect anomalies in GOOSE messages.

In literature, the available solutions for securing GOOSE messages are either IT based, or OT based. However, there is a need for developing holistic solutions which involve both IT and OT domains. In this regard, this paper proposes a holistic solution for securing GOOSE messages using a sequence content resolver which combines both IT and OT based solutions. On IT side, MAC value is checked to confirm the integrity of the received message, then a strategy based on transmission sequence counter sqNum and event update counter stNum is devised to introspect the sequence and content of GOOSE packets. On OT side, once it is confirmed that there is change in data content, confirmation is acquired from the neighboring IEDs and the counterfeit messages are segregated from the real ones based on a rule based applied security. Table 1 presents the qualitative feature comparison of the proposed holistic solution with the existing solutions to secure GOOSE messages. The effectiveness of the proposed holistic solution is demonstrated by conducting performance evaluation tests on the real-time cyber-physical test bed of a standard microgrid. The main research problem is to simulate cyberattacks (FDI attacks, mainly masquerade and replay attacks) on GOOSE protocol using real time digital simulator on a standard microgrid and later deploy mitigation technique to counter these attacks. Hence, the main contributions of this work are as follows:

1. Developed real time test bed for studying cyberattack (FDI) on GOOSE protocol using RTDS and Snort.
2. Proposed a novel IT + OT security scheme for securing GOOSE protocol. Snort is used to inject FDI attacks and Wireshark is used to monitor the GOOSE packets. An anti-Snort is proposed by the name of sequence content resolver (SCR) which nullifies the impact of Snort. SCR comprises of two modules i.e. COMM and ELEC, the former is the communication module which deals with the replay attacks (sequence of GOOSE packets) and the latter is the electrical module which deals with the masquerade attacks (content of GOOSE packets). Both modules together constitute SCR and mitigate the FDI attacks.
3. Demonstration of cyberattacks and proposed mitigation strategy on a real time digital platform. The result of masquerade attack is presented to create a system fault alike situation on power system. The exploited GOOSE packets effect the protection and control (P&C) IEDs which trip the breakers or generate islanding scenario in case of microgrid, this impact is evaluated, discussed and presented.

**Table 1:** Cybersecurity solutions for securing GOOSE message.

	IT		OT/machine learning	IT + OT based deterministic
	Authentication	Encryption		
Hussain et al.^19^	✓	✗	✗	✗
Hong et al.^15^	✗	✗	✓	✗
Ustun et al.^20^	✗	✗	✓	✗
Wang et al.^17^	✗	✗	✓	✗
Rodríguez et al.^21^	✓	✓	✗	✗
This work	✗	✗	✗	✓

The rest of the paper is organized as follows. “IEC 61850 protocols and control authority” presents the background of IEC 61850 standard and control authority. “Methodology to validate cyberattacks” discusses the development of testbed and demonstration impact of cyberattacks. “Implementation of hybrid solution” discusses the design and implementation of the proposed holistic sequence content resolver for mitigation of cyberattacks on GOOSE messages. Finally, conclusions are presented in “Conclusions”.

## IEC 61850 protocols and control authority

The first edition of IEC 61850 was initially developed for substation automation systems. In the later editions of IEC 61850 standard, it was extended to entire power utility automation systems. The IEC 61850 standard defines four protocols namely GOOSE, SV, MMS and SNTP:

- GOOSE for switching signals from IEDs to circuit breakers (CBs);
- SV for measurement values from merging units (MU) to IEDs;
- Manufacturing message specification (MMS) to exchange measurement readings and control commands between human–machine interface (HMI) and IEDs;
- Simple network time protocols (SNTP) for time synchronization of IEDs with GPS master clock.

**Figure 1:**
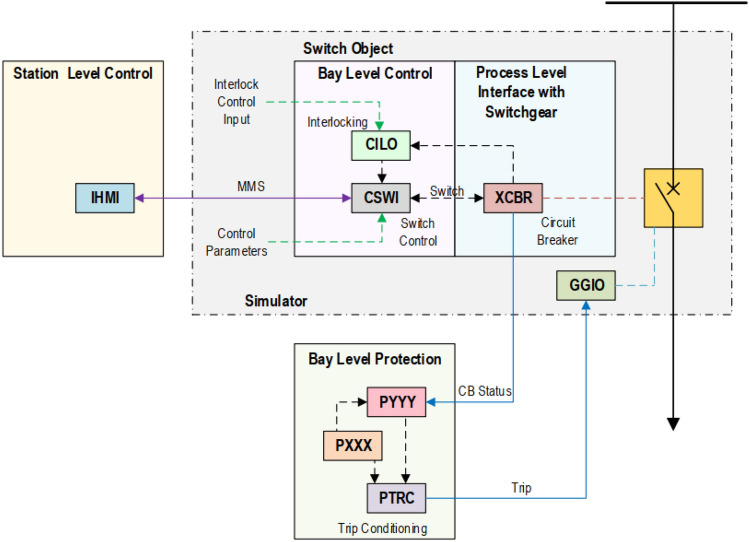
Circuit breaker control based on switch object^22^.

An operator can trip circuit breakers via GOOSE messages during fault or maintenance. To grant access to operators at different locations and to avoid conflicts between them, a concept called control authority is used, which designates an operator’s right to switch a specific circuit breaker^22^. This implementation is based on an entity called a switch object (SO), which is a combination of three logical node (LN) instances, XCBR (or XSWI), CSWI and CILO as shown in Table 2 and Fig. 1. A SO takes the control parameters and an interlock logic as inputs. A particular SO can be mapped to the desired circuit switch in the simulation for control operations. A remote client can access the SO for control purposes using the MMS protocol as shown in Table 3. The binding of external trip signals (published as GOOSE messages) to the corresponding circuit breaker is achieved using a generic input (GGIO LN instance), and done independently from the SO.

**Table 2:** Logical node classes and control parameters as per IEC-61850^6^.

	Description
**Logical node class (IEC 61850-7-4)**	
XCBR	Circuit breakers—switches with short circuit breaking capability
XSWI	Circuit switches—switches without short circuit breaking capability
CSWI	Switch controller—control all switching conditions above process level
CILO	Interlocking function—enable a switching operation if interlocking conditions are met
**Control parameter**	
XCBR/XSWI.Loc	Represents the status of an actual switch at the process and allows taking over the manual control authority
LLNO.MltLev	Enables for more than one originator to hold control authority at the same time
CSWI.Loc	Represents the control behavior of the logical node (bay level)
CSWI.LocSta	Represents the switching authority at the station level

**Table 3:** Switchgear control based on control authority^6^.

Control parameters				Control authority at each level			
Switch	Bay control			Manual control	Originator category (OrCat)		
XCBR.Loc XSWI.Loc	LLNO.MltLev	CSWI.Loc	CSWI.LocSta	Process^a^	Bay^b^	Station^c^	Remote^c^
TRUE	FALSE	Not applicable	Not applicable	Always allowed	Not allowed	Not allowed	Not allowed
FALSE	FALSE	TRUE	Not applicable	Always allowed	Always allowed	Not allowed	Not allowed
FALSE	FALSE	FALSE	TRUE	Always allowed	Not allowed	Always allowed	Not allowed
FALSE	FALSE	FALSE	FALSE	Always allowed	Not allowed	Not allowed	Always allowed
TRUE	TRUE	Not applicable	Not applicable	Always allowed	Not allowed	Not allowed	Not allowed
FALSE	TRUE	TRUE	Not applicable	Always allowed	Always allowed	Not allowed	Not allowed
FALSE	TRUE	FALSE	TRUE	Always allowed	Always allowed	Always allowed	Not allowed
FALSE	TRUE	FALSE	FALSE	Always allowed	Always allowed	Always allowed	Always allowed

^a^Current and voltage transformers (CT/VT) connected to MU.

^b^Switch controller communicating at process level with MU via SV and CB via GOOSE and MMS.

^c^Communication with switch controller via MMS.

## Methodology to validate cyberattacks

A testbed is developed to create cyberattacks or false data injection (FDI) attacks on power systems using real time digital simulator (RTDS). It can be further utilized to investigate the attacks on IEC 61850 communication protocols and to analyse its impact on power systems.

### Testbed for implementation and modification of IEC 61850 communication

The time stringent communication protocols in IEC 61850 are GOOSE and SV. The GOOSE is responsible to send control commands from P&C IEDs to circuit breakers (CBs) IEDs, while the SV provides sampled and digitalized values of current and voltage measurements to the same P&C IEDs from merging units (MU). Hence, both these control commands and measurements data being transferred by GOOSE and SV protocol respectively fall under the protection scheme of substations where timely measures are necessary. An attacker who can exploit the vulnerabilities of these protocols can do great damage both to power equipment and supply being fed to consumers. The modification in SV packets leads P&C IEDs to receive fake data and based on this fake data they directly or on the approval of operator can issue wrong commands to associated CBs. In addition to this indirect attack via SV to change the status of the CB, the attacker can also directly control GOOSE packets to trip/reclose CBs of his choice to demonstrate a picture of fear, havoc and economic turmoil among the working personnel and connected customers. The explained attack is conducted in following two steps:

1. Real time simulation of GOOSE packets between IEDs,
2. We feed fake data to IEDs through the GOOSE protocol, simulating a compromised IED accessed and controlled by an attacker.

**Figure 2:**
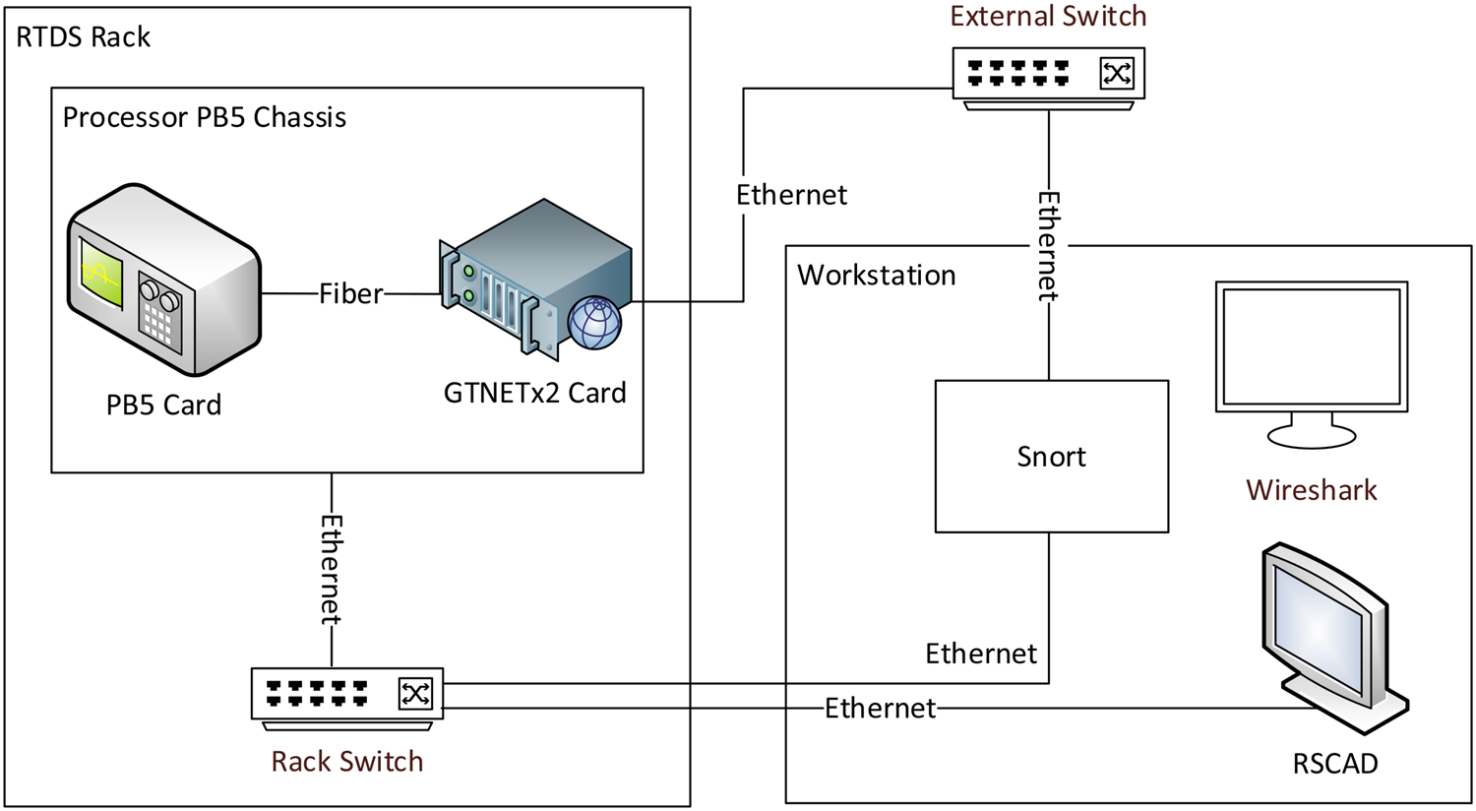
Testbed with RTDS, Snort, and Wireshark^6^.

The implementation is carried out using an interconnection system of RTDS^23^, Wireshark^24^ and Snort^25^ as shown in Fig. 2. The first system provides real time simulation features for any power system to be studied. Its recent network interface card GTNETx2 is the communication interface for simulation of communication packets coming out and going into the simulated power system. Each GTNETx2 card has two modules, and each can simulate one protocol at a time such as GOOSE, SV, MMS, DNP3 etc. The setup to test and modify the simulation of communication packets is through a publisher-subscriber setup where transmission is broadcasted in multicast fashion by the publisher and different subscribers can subscribe to the data being transmitted. Due to multicast nature of GOOSE packets, an attacker who gets access to the substation’s network can view the GOOSE packets and also inject counterfeit GOOSE messages with faulty information. This action may lead to tripping or holding the circuit breakers which damages the equipment causing harm to the stable operation of power system. The GOOSE messages being published and later being subscribed by particular IEDs are monitored by an open-source tool Wireshark.

For modifying the packets, Snort, another open source tool, with some changes is being utilized to capture the packets from publisher, modify them and later inject them in to the network. Snort basically captures the GOOSE packets published by P&C IEDs and modifies them by changing the value of stNum field to high number and value of data field as selected by the user. These modified packets are then re-published by Snort which are received by the CB IEDs. The testbed has the advantage that it is based on open-source tools. It is capable of simulating and modifying the communication packets of various protocols such as GOOSE, SV etc. which constitute an attack and is later supporting in evaluating the impact of modification in communication packets over the automated power system.

### Simulation and modification of GOOSE packets

**Figure 3:**
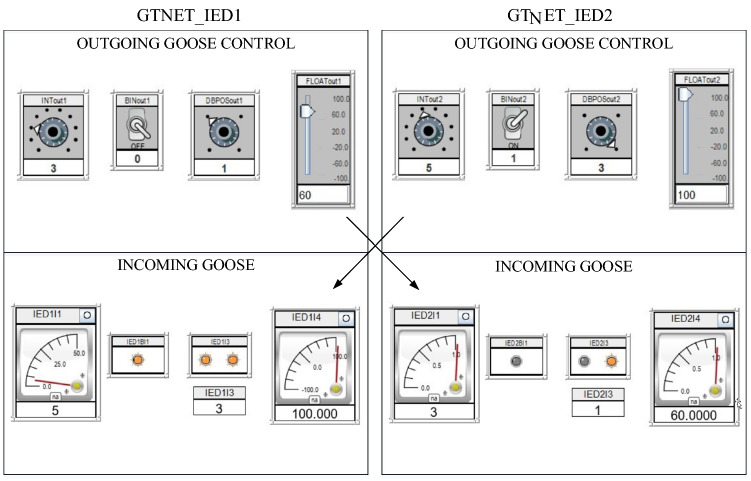
RTDS runtime for GOOSE communication between IED 1 and IED 2 before the manipulation of packets by the attacker^6^.

**Figure 4:**
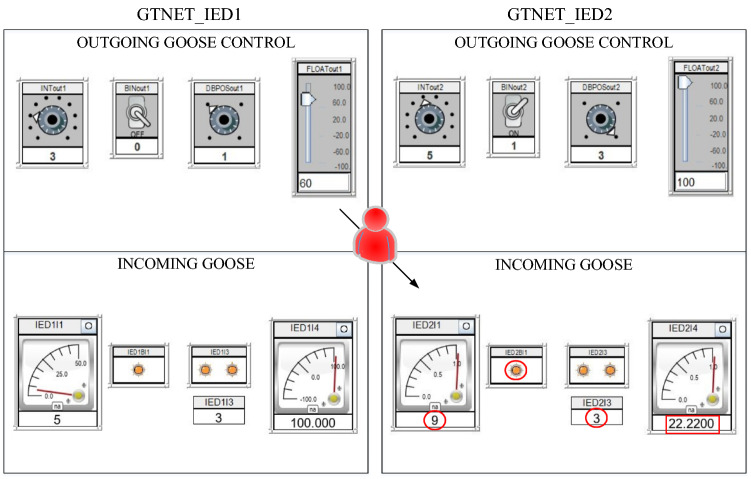
RTDS runtime for GOOSE communication between IED 1 and IED 2 after the manipulation of packets by the attacker^6^.

GOOSE protection is the most critical protocol as it is used in protection schemes to trip/reclose CBs in response time matching within 3 ms. In order to simulate the GOOSE packets between publisher–subscriber setup, we have engaged both modules of a GTNETx2 card; one acting as sender or publisher while the other is behaving as receiver or subscriber. The communication packets in between them are of 4 different data types (integer, binary, two-bits and floating point) out of which the tripping/reclosing command is usually sent with Boolean type of data. As shown in Fig. 3, IED 1 is sending the 4 types of data [3 0 1 60] and same is subscribed by IED 2 while IED 2’s broadcasted data [5 1 3 100] is being subscribed by IED 1. The attack is simulated in Fig. 4 when IED 1 is acting as publisher with data [3 0 1 60] which is being lost and modified because IED 2 is receiving counterfeit data [9 1 3 22.22]. This modification is conducted by capturing the publisher packets using Snort with GOOSE packets important parameters i.e. control block (gocbRef) and data set (datSet). The packets are monitored on Wireshark and this experiment validates the direct FDI attack on GOOSE communication between IEDs and its impact on electrical side is very harmful. For instance, an attacker can corrupt the Boolean value in order to open circuit breakers for cascaded tripping affecting consumers or he can also keep the breakers in closed position during actual system fault to damage the equipment.

**Figure 5:**
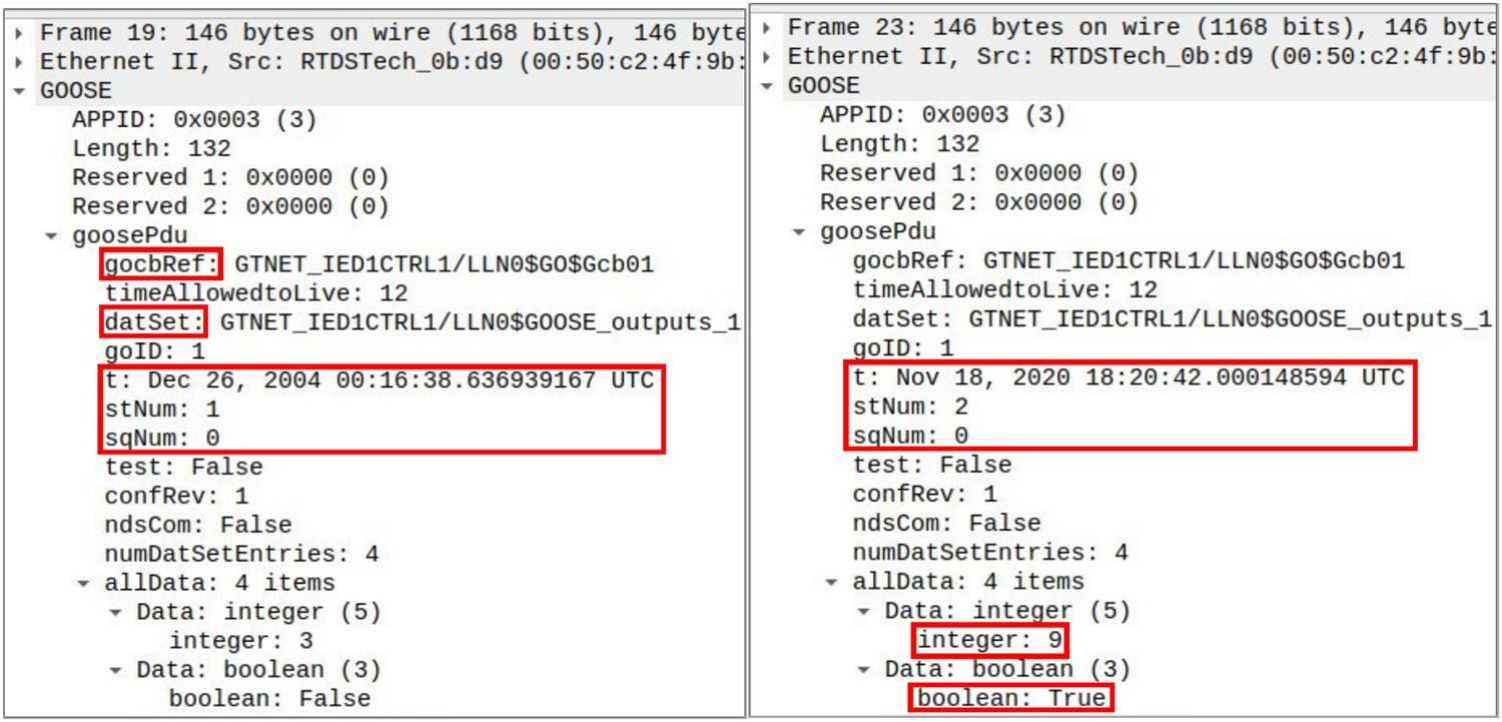
Original and counterfeit GOOSE packets for IED 1 on LAN port^6^.

The original GOOSE packet and the corresponding modified packet as observed in Wireshark is shown in Fig. 5. The modification is done by the attacker in all 4 data types and IED 2 is receiving the counterfeit message [9 1 3 22.22] instead of originally broadcasted message [3 0 1 60] by IED 1 as publisher. The packets are focused on the integer and Boolean data types only as the Boolean data is normally used to change the status of the breaker. The two counters i.e. status (stNum) and sequence (sqNum) in GOOSE packets are to be carefully observed from security perspective because the first status counter increments on every new event or status change while the latter sequence counter increments on periodic transmission of repetitive packets. sqNum keeps on increasing its value by 1 until its maximum value is reached while stNum will stay as it is and will only change once there is any new event meaning once there is any change in the data items of GOOSE packets. The original and counterfeit messages can be compared in parallel using their timestamps as the genuine packet originated from GTNETx2 card has older timestamp of year 2004 which can be synchronized to present date and time but for identification purposes of original GOOSE packets, we did not synchronize the time and date. The timestamp of fake packet is aligned with the time of experiment i.e. year 2020 as per the workstation’s clock with Snort installation used for modification of packets.

The GOOSE protocol is the critical one among others in IEC-61850 power system automation standard due to its role in protection schemes of electrical network, hence suitable countermeasures should be devised based on the concepts of cyber and physical domains to secure power system communication^26^.

### Impact of FDI attacks on simple and complex electrical systems

**Figure 6:**
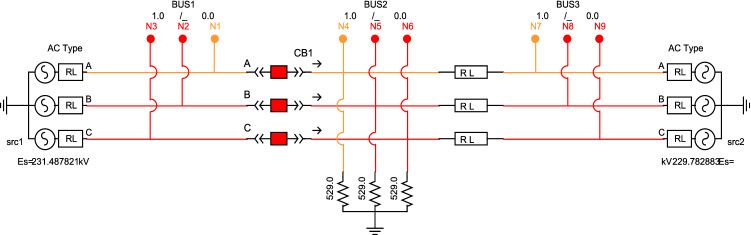
3-phase doubly fed system in RSCAD Draft with 3 buses, circuit breaker, isolators and load^6^.

In a doubly fed system with 3 buses as shown in Fig. 6; bus 1 and 2 has a circuit breaker CB1 in the middle and there is resistive load connected to bus 2 while bus 3 is connected directly to bus 2 with line impedance. The bus 1 and 3 are source buses. The GOOSE packets can send tripping/reclosing command to CB1 and its impact is evaluated on electrical side. Normally, the circuit breaker is closed but to disturb the system, a GOOSE tripping command can be sent as discussed before by changing the data item of the Boolean type to TRUE. This will cut off Source 1 on the left side and Source 2 on the right side will be the only one remaining now feeding the resistive load at bus 2. The redundancy of dual source has been compromised, the breaker current will drop to near zero while the condition of bus voltages before and after tripping is given in Table 4.

**Table 4:** Logical node classes and control parameters as per IEC-61850.

Parameters	*Source bus 1* (kV)	*Load bus 2* (kV)	*Source bus 3* (kV)
*Voltage pre-tripping*	230	229.9	230
*Voltage post-tripping*	231.5	226.8	228.7

**Figure 7:**
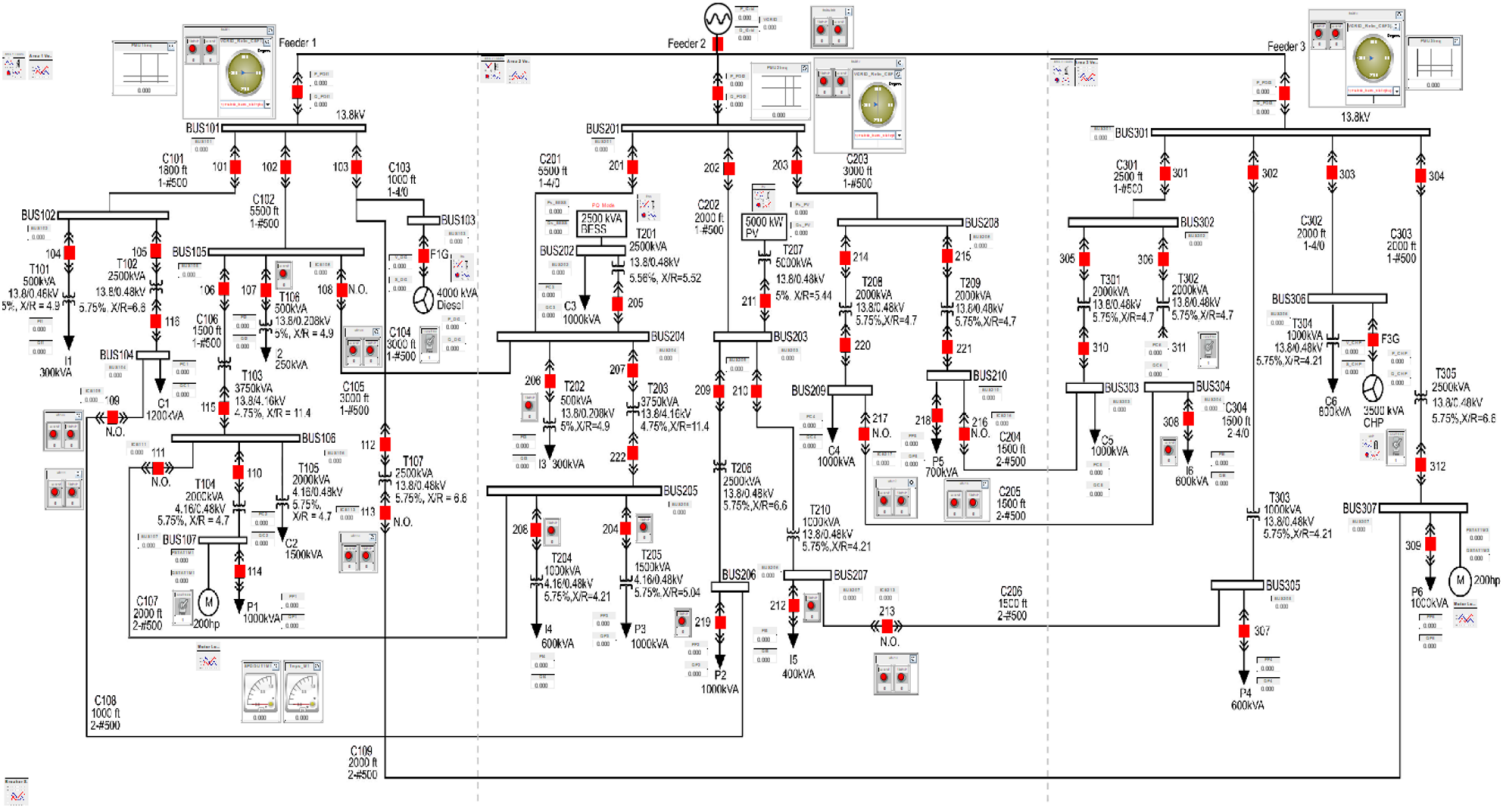
Runtime single line diagram of Banshee microgrid.

This impact on a simple electrical system creates disturbance and stability issue once the circuit breakers are controlled by counterfeit GOOSE commands. The effect becomes manifold as the circuit becomes large and complex. We have simulated and modified the GOOSE packets and now we will discuss its impact on a standard electrical system known as Banshee microgrid as shown in Fig. 7^27^.

Banshee microgrid, a commonly used system in real time simulation studies, includes three radial feeders connected to the grid that feed three independent areas. The independent areas can work autonomously in islanding mode but can also be inter-connected through normally opened (N.O.) tie switches. Switching between grid-connected and islanding mode simply requires to trip the main breaker of the area. This can be achieved for example by sending a simple GOOSE packet with Boolean data set to 1 that triggers the islanding mode. When entering islanding mode, the frequency drops. If the generation in the independent area, now isolated from the grid, is insufficient to satisfy the load demand in isolated area, further tripping or load shedding may follow. Isolating power components by tripping specific breakers in different areas, eventually leads to disturbances in the overall system.

Regarding the generation assets, the Banshee microgrid includes^6^:

- In the first area, a 4 MVA diesel that implements the governor, exciter and synchronous generator model.
- In the second area, a 5 MVA photovoltaic (PV) with 2.5 MVA battery energy storage system (BESS) based on the average value model for converter.
- In the last area, a 3.5 MVA natural gas fired combined heat and power (CHP) that implements the same governor, exciter and synchronous generator model used in area 1. The system further includes the following components:
- Transformers: with primary voltage level of 13.8 kV stepping down to 4.16 kV, 480 V and 208 V secondary voltage levels.
- Loads: dynamic aggregated ones (categorized into critical, priority and interruptible) and motor loads (induction motor driving 200 horsepower (hp) chiller compressor).
- Cables: modelled with series resistance-inductance (RL) impedances.
- Circuit breakers: including synchro-check capability for main incomers (3 areas) used to connect each area to the grid. All these breakers including in each area can be controlled by external trip/reclose signals or manual push buttons in Runtime of RSCAD.

**Figure 8:**
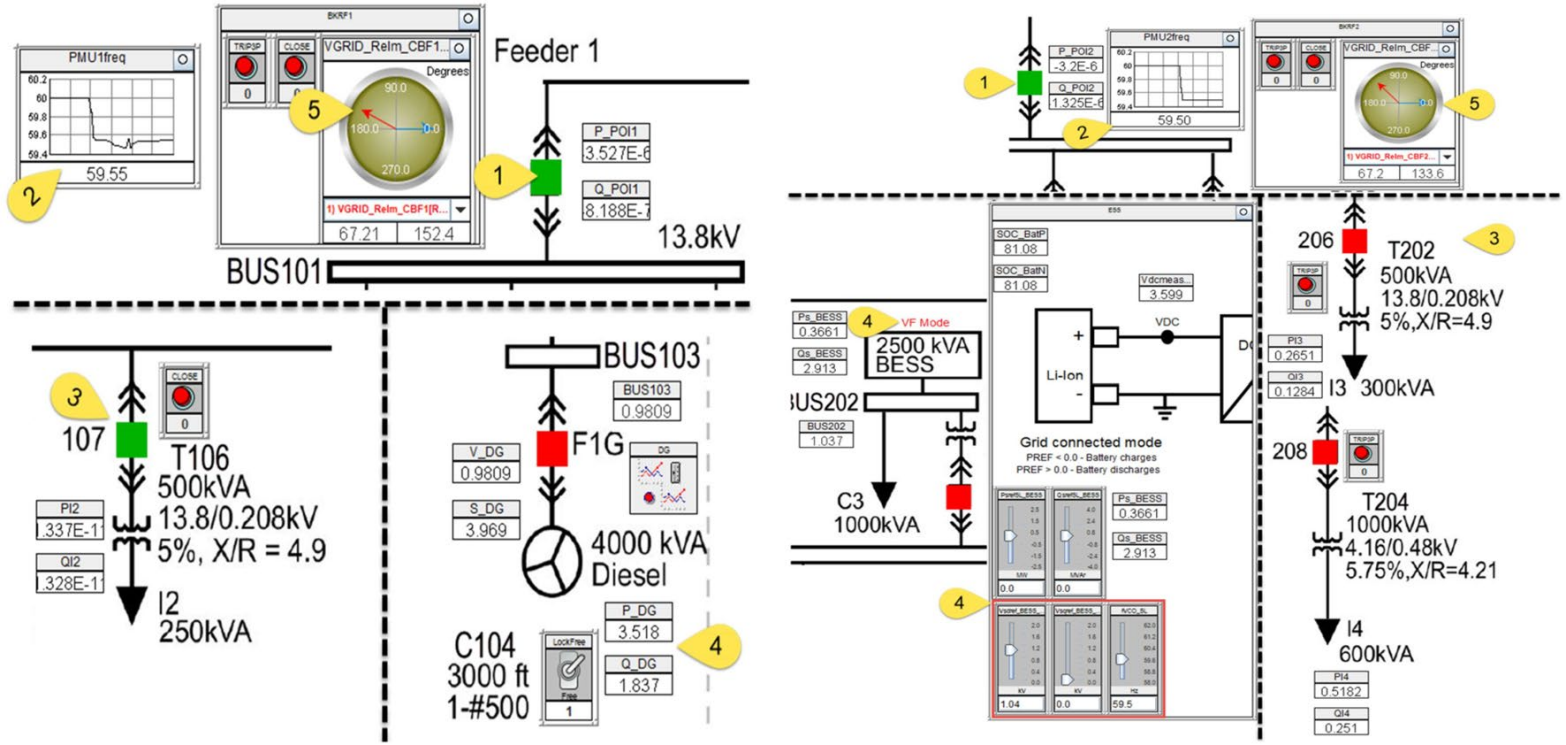
Islanding Area 1 or Area 3 vs. Area 2^6^.

Due to its design, the Banshee microgrid is a great fit to investigate islanding scenarios. In such scenario, an area is islanded to make sure that its frequency would remain stable, and that generation can keep up with the demand in the area at least. In case of the frequency drops, each area has controls in place to prioritize some loads and shed them if needed. Islanding also modifies the generation assets operating points; for instance, BESS in area 2 shifts from PQ to VF mode. Figure 8 shows the difference in steps 1 to 5 observed after islanding between areas with renewable generation (area 2) and areas with conventional generation (areas 1 and 3). After islanding an area by tripping the main incomer breaker, the frequency drops below the nominal frequency and the area has not enough generation capacity to reach nominal frequency back. Thus interruptible loads are shed, such as I2 in area 1. Furthermore, the sources change to new operating points and finally the rotating phasors at the top of each diagram indicate the discrepancy of voltage frequency between the grid and the area. In area 2, the situation is more favourable as the battery provides power to the area allowing it to avoid tripping interruptible loads such as I3 in area 2.

Beyond real time islanding scenarios, the Banshee microgrid provides the opportunity for power system studies for many electrical systems cyberattacks. For instance, an attacker injecting false data between the Aggregator (an equipment responsible for communication with distributed energy resources (DERs) and optimization to provide economical energy from them) and DERs could prioritize specific DERs thus creating monopoly for selling electricity or corrupt load shedding controls. Denial of Service (DoS) attack can also efficiently target one of the Aggregator to block available power information from DERs, resulting in generation assets to overload for long time, leading to their damage or failure.

## Implementation of hybrid solution

In this work, we proposed a hybrid solution that functions on knowledge of cyber and physical domains of the power system. ICT-based solutions may present a high false negative/positive ratio and cannot do much protection once the attacker breaches the electronic boundary of substations. Hence, holistic countermeasures based upon communication and power fields provide enhance security towards the cyberattacks. The communication packets and protocols as defined in IEC-61850 for power system communication consists of header and payload. An attacker tries to exploit either or both in order to launch replay and masquerade attacks. Hence, the proposed solution tackles both sequence and content of the packets and handles them to rule out traces of exploitation in terms of false data injection.

The sequence will be checked by the first module based on communication concepts, and the content will then be investigated by the second module based on electrical concepts. For GOOSE communication packets, the sequence can be checked by analyzing the status and sequence counters (stNum and sqNum). The replay attacks can be detected and mitigated if these counters contain older values compared to the previously stored packet. In addition, timestamps can be additionally used to check in the case if the attacker has replayed an older packet instead of creating a new one with new values of the counters. For content exploitation, we have to address the electrical understanding of the data items in GOOSE packets. As the data items contain mostly binary values representing the tripping/reclosing status of circuit breakers, hence we have to devise or adopt a method to get a valid confirmation of such requests coming from protection and control (P&C) IEDs to the CB IEDs.

**Figure 9:**
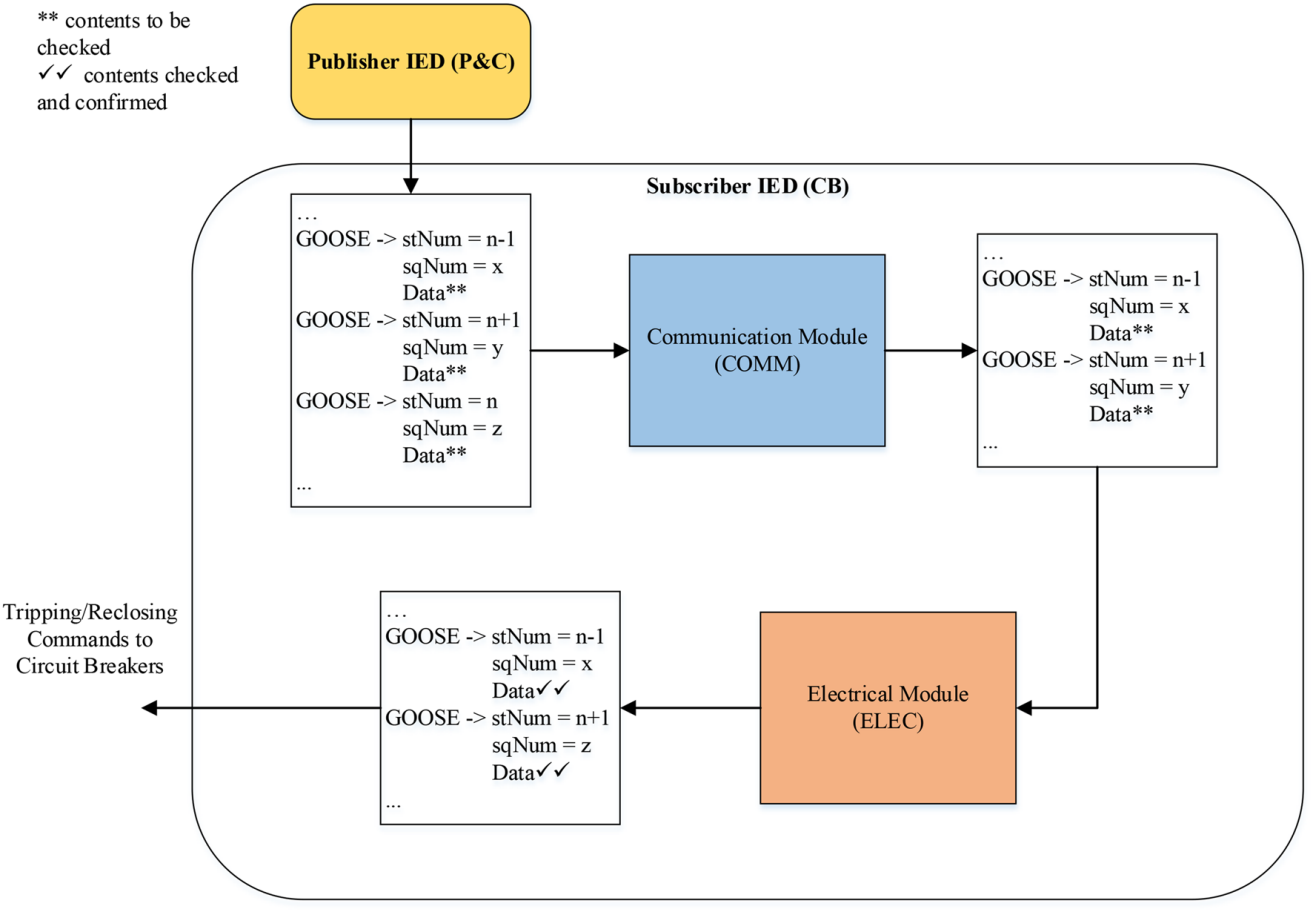
Block diagram of sequence content resolver (hybrid solution).

In^15^, authors proposed a scheme to check changes in relay settings and sensor measurements and controlling directly CB IEDs by electrical based mathematical equations and calculations. Based on these calculations, they check the behavior of other IEDs in the vicinity compared to the target IED and await their approval to honor or dismiss such requests, resulting in changing the breaker status. The block diagram of the mitigation strategy is shown in Fig. 9. In addition to this strategy and to make our solution effective, the use of MAC algorithms on communication level is also applied to authenticate the data and source of communication packets^21^. The publisher IED appends the GOOSE message with MAC value generated using the secret pre-shared key and sends it to the subscriber. The subscriber IED receives both the GOOSE message and MAC value. Then subscriber recalculates the MAC value for the received GOOSE message using the secret pre-shared key and compares this calculated MAC value with the received MAC value. If the MAC values do not match, the packet is rejected.

**Figure 10:**
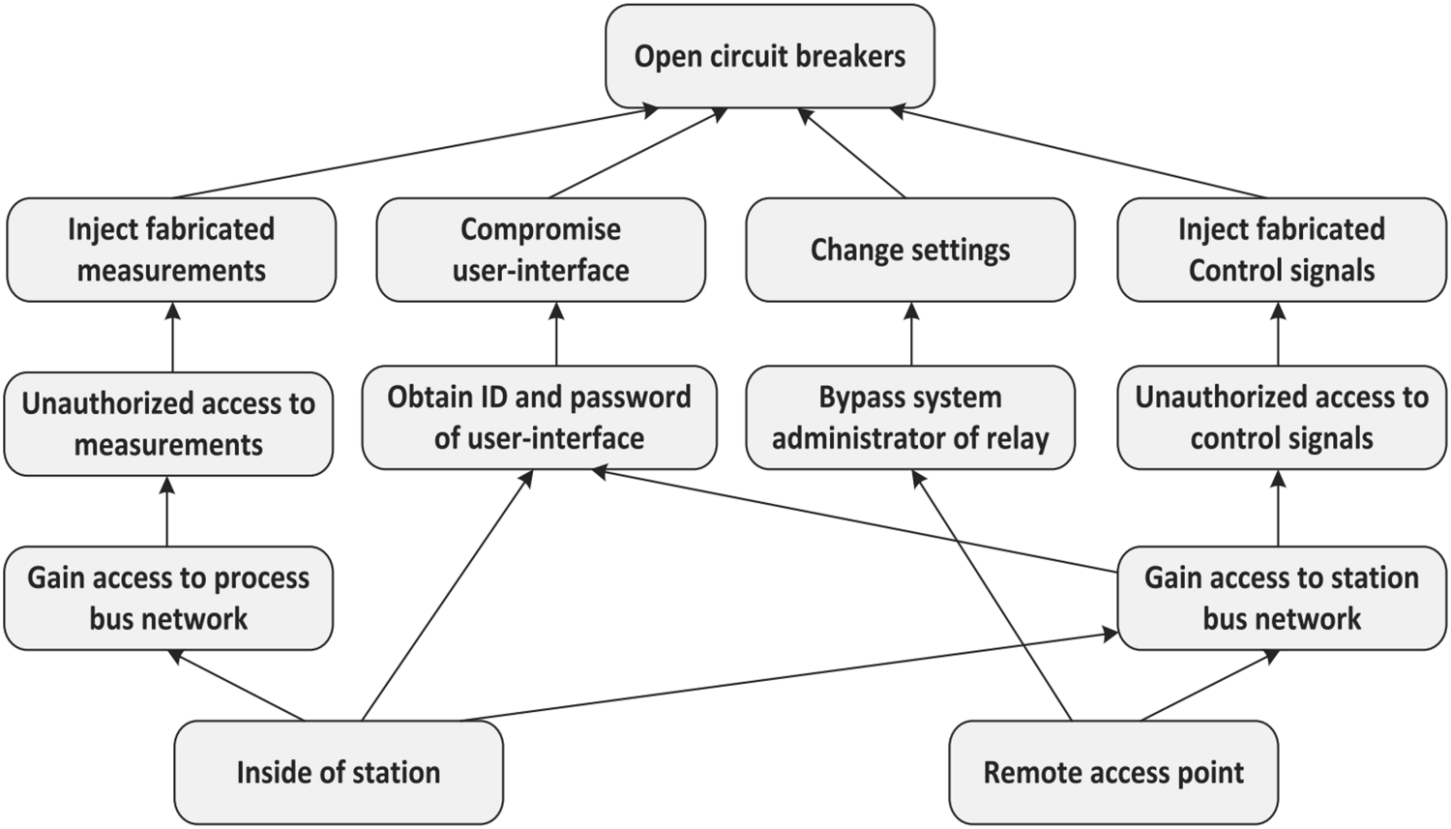
Attack tree showing the paths for potential cyberattacks^15^.

The sequence module in the sequence content resolver will check stNum and sqNum to decide whether it is a replay attack or not, while the content module will rule out masquerade attack by cross-validating the commands with neighboring IEDs. The try to falsely open or close circuit breakers can be via different paths as described in an attack tree as shown in Fig. 10^15^. The access to the substation network can be from inside the substation (process bus) or remotely from outside (station bus). Afterward, an attacker would try to access HMI, relays settings, control commands, and sensor measurements either individually or in combination, all of this in an attempt to trip or reclose circuit breakers. The intended impact is to trip circuit breakers, transmission lines, bus bars, transformers, and other critical infrastructure providing either supply to consumers or protection to the infrastructure. The objective is to create a havoc in the working personnel and the connected consumers in order to create an economic turmoil.

Mainly, three types of exploitations are attempted by an attacker^15^ to disrupt the operations of circuit breakers. The first one is to change the configuration settings of relays. The second is to inject false data into measurement sensors. The third is to directly control the circuit breakers to affect the connected consumers for malicious objectives. The first two exploitations are indirect and lead to tripping/reclosing of circuit breakers, while the third one is the direct attempt to change breaker status by issuing counterfeit messages. In^15^, they have successfully developed the method to counter-check the control commands with surrounding IEDs. Only after their approval will allow or block tripping/reclosing signals be put through. Our content module has adopted the physical or power domain-based mitigation methods from^5^. The content of GOOSE packets is verified by those techniques, resulting in passing the genuine commands and blocking the ones originating from an attacker. The difference in our work compared to^5^ is that we have provided a holistic cyber-physical solution at the subscriber IED level. The cyberattack will be fought on both levels of communication and power domains by the proposed novel sequence content resolver.

**Figure 11:**
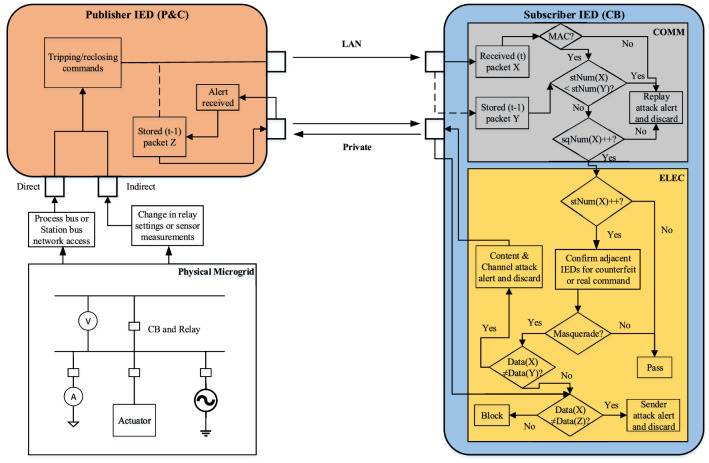
Functional diagram of novel sequence content resolver for GOOSE communication packets.

Figure 11 shows the functional diagram of the novel sequence content resolver. The publisher or P&C IED can be accessed directly from the substation network by process or station bus in order to change the status of circuit breakers. The indirect access would be to again open/close the circuit breakers by changing the settings of relays or sensor measurements such as current/voltage transformers and merging units. The control commands are sent by P&C IEDs to CB IEDs and the previous packets (Z) are stored in order to communicate privately in case of attack on channel between the publisher and subscriber IED and later they help too to diagnose the attack on publisher IED. Once the packets are received by subscriber IED over LAN, their previous counterparts (Y) are stored, and the present packets (X) are submitted to the communication module (COMM), which will check the sequence of packets based on stNum and sqNum counters. The function of this module is to look out for replay attacks by issuing a proper alert. For replay attacks, the stNum and sqNum of present packets are compared with that of their previous counterparts, if they are older, then the packet is discarded with an alarm of replay attack. Otherwise, the packets are passed to the electrical module (ELEC) for investigation of masquerade attacks or content exploitation. The increment in stNum is analyzed with the help of neighboring IEDs in order to differentiate between real and fake commands. After getting approval from surrounding IEDs, the real commands are passed while fake commands are alarmed as masquerade attacks. Afterward, only the data items of the packets with their past counterparts both in publisher and subscriber IEDs are checked step by step to find and declare attacks on sender and channel, respectively. Further description on the working of both modules is given below:

### Sequence or communication module (COMM)

This module checks for the exploitation in the sequence in the form of replay attacks. First, the MAC value is calculated for the received message using the pre-shared secret key and compared with the received MAC value. If the MAC value does not match the packet, it is discarded, otherwise the packet is processed further. If the value of sqNum is lesser than that of the previous packet or the timestamp is two minutes older^21^, it means that the attacker is replaying an old packet, and it should be discarded. The same thing is done for a packet with older stNum but if the value of stNum is greater than that of its previous packet, it can be either a genuine status change or a masquerade attack by the attacker. It also signals that the data items of the GOOSE packet are now different than that of the previous packet, and hence it should be analyzed by the content module.

### Content or electrical module (ELEC)

In this module, the packets are investigated for masquerade attacks where the contents of the packets are definitely changed by the attacker. The change is signified by increased stNum, but now the module will take surrounding IEDs into confidence for confirmation of this command signal. The IEDs are approached by calculating different parameters depending upon the case of exploitation, as explained in^15^, and the same is adopted in this work. For a change in relay settings or sensor measurements, they indirectly impact the associated control commands of circuit breakers and hence should be verified by the neighboring IEDs before executing it. The direct attack on circuit breaker control via the GOOSE command is also possible and can be carried out by the attacker. The following parameters and calculations in time less than operational protection scheme is investigated for target IED by communicating the neighboring IEDs^15^:

1. For a change in relay settings, if there is no loss of protection coordination scheme, the adjacent IEDs will permit control command; otherwise, it will be blocked.
2. For a change in sensor measurements, the fault transients of neighboring nodes will be compared, and the decision to permit or block the control command by the target IED will be taken.
3. For direct circuit breaker control attack on IED, the security gateway will respond if there is an impact with respect to line overloading and bus voltage conditions. If the impact is potential cascaded tripping of lines or voltage stability issue, then the blocking signal will be sent by the security gateway to the IED under consideration.

**Figure 12:**
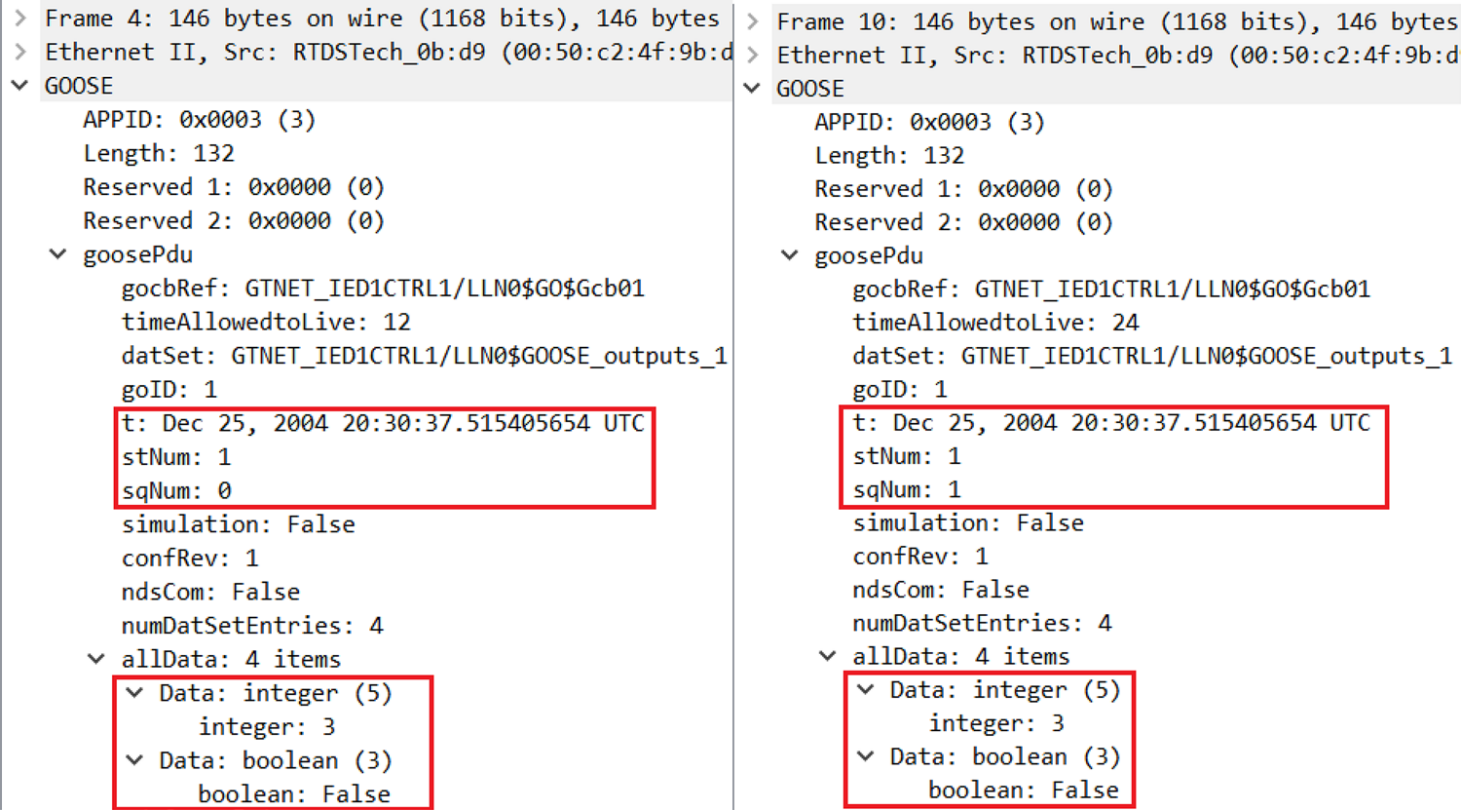
Original GOOSE packets for IED 1 on LAN port with sequence content resolver.

After the installation of sequence content resolver at subscriber IED level, the genuine packets are streamlined in contrast to "Simulation and modification of GOOSE packets" and Fig. 5. The counterfeit messages which were sent by the attacker with incremented stNum and changed data items are discarded while the real and genuine packets are passed with the same stNum and incremented sqNum as shown in Fig. 12. Further from the comparison of fixed timestamps, it is also clear that both packets are originated from the network interface card of RTDS. Therefore, both timestamps are the same representing the same fact, and are set so for easy identification of genuine packets, while fake messages contain the timestamps of the workstation from where they are originated. Moreover, the stNum will be incremented in case of any new event or status change in case of fault or maintenance and that will be passed with the approval of neighboring IEDs. Otherwise, the stNum will remain the same (1–1), and sqNum will be only incremented (0–1) to show the transmission sequence of packets. Any modification in the contents of packets in the name of a new event (incremented stNum) will be flagged and discarded or blocked on the spot.

## Conclusion

This work deals with a methodology to validate cyberattacks on GOOSE protocol and later evaluate their impact on power systems. This is achieved by developing a testbed focusing on GOOSE in which GOOSE messages are implemented, modified and later sent to a simulated electrical system. The impact of these modified messages are evaluated to devise a novel rule based cybersecurity solution by the name of Sequence Content Resolver. Future work will cover other protocols such as SV and MMS and we will investigate the application of artificial intelligence and machine learning techniques to develop cybersecurity solutions using and enhancing the same developed testbed.

## Data Availability

All data generated or analyzed during this study are included in this published article.

## Abbreviations

BESS: Battery energy storage system
CB: Circuit breaker
CHP: Combined heat and power
CT: Current transformer
DER: Distributed energy resources
DoS: Denial of service
ECDSA: Elliptic curve digital signature algorithm
FDI: False data injection
GOOSE: Generic object-oriented substation events
HMI: Human–machine interface
hp: Horsepower
ICT: Information and communication technologies
IEDs: Intelligent electronic devices
IT: Information technology
LN: Logical node
MAC: Message authentication code
MMS: Manufacturing message specification
MU: Merging unit
N. O.: Normally opened
OT: Operational technology
P&C: Protection and control
PV: Photovoltaic
RL: Resistance–inductance
RTDS: Real time digital simulator
RSA: Rivest–Shamir–Adleman
RSS: Rainbow signature scheme
SNTP: Simple network time protocol
SO: Switch object
SV: Sampled value
VT: Voltage transformer
